# Insights into the multifaceted role of circular RNAs: implications for Parkinson’s disease pathogenesis and diagnosis

**DOI:** 10.1038/s41531-021-00265-9

**Published:** 2022-01-10

**Authors:** Epaminondas Doxakis

**Affiliations:** Center of Basic Research, Biomedical Research Foundation, Academy of Athens, 11527 Athens, Greece

**Keywords:** Parkinson's disease, Parkinson's disease

## Abstract

Parkinson’s disease (PD) is a complex, age-related, neurodegenerative disease whose etiology, pathology, and clinical manifestations remain incompletely understood. As a result, care focuses primarily on symptoms relief. Circular RNAs (circRNAs) are a large class of mostly noncoding RNAs that accumulate with aging in the brain and are increasingly shown to regulate all aspects of neuronal and glial development and function. They are generated by the spliceosome through the backsplicing of linear RNA. Although their biological role remains largely unknown, they have been shown to regulate transcription and splicing, act as decoys for microRNAs and RNA binding proteins, used as templates for translation, and serve as scaffolding platforms for signaling components. Considering that they are stable, diverse, and detectable in easily accessible biofluids, they are deemed promising biomarkers for diagnosing diseases. CircRNAs are differentially expressed in the brain of patients with PD, and growing evidence suggests that they regulate PD pathogenetic processes. Here, the biogenesis, expression, degradation, and detection of circRNAs, as well as their proposed functions, are reviewed. Thereafter, research linking circRNAs to PD-related processes, including aging, alpha-synuclein dysregulation, neuroinflammation, and oxidative stress is highlighted, followed by recent evidence for their use as prognostic and diagnostic biomarkers for PD.

## Introduction

Parkinson’s disease (PD) is the second most common neurological disorder affecting about 1% of people over 60. It is characterized by resting tremor, rigidity, and bradykinesia. In addition, patients with PD also experience a spectrum of non-motor symptoms such as autonomic disturbances, impaired cognition, depression, and sleep disturbances^1^. The neuropathological hallmark of PD is the loss of dopaminergic neurons and the presence of fibrillary aggregates known as Lewy bodies and Lewy neurites in the substantia nigra pars compacta (SNpc) and other brain regions^2^. The etiology of PD is complex and is believed to arise from the interaction of genes, environmental factors, and aging. Although genetic causes have provided invaluable insights into the pathogenesis, they represent the minority of cases, with the remaining likely resulting from epigenetic changes caused by environmental risk factors and aging^3^.

Circular RNAs (circRNAs) are a newly recognized class of single-stranded regulatory RNAs formed by head-to-tail splicing (backsplicing) in which a downstream 5′ splice site is covalently connected to an upstream 3′ splice site of an RNA molecule. The result is an enclosed non-polyadenylated circular transcript^4–6^. Due to the lack of free ends typically targeted by 3′ and 5′ exoribonucleases, circRNAs are highly stable with a half-life of more than 48 h compared to ~6 h of linear transcripts^7,8^. The length of circRNAs is heterogeneous, ranging from approximately 100 to 10,000 nucleotides, and for most genes, the amount of circRNAs is between 0.1 and 10% of the linear amounts, with most being <1%^7^. CircRNAs are widely expressed in eukaryotic cells and exhibit cell-, tissue-, and developmental stage-specificity suggesting they have regulatory functions in different biological processes^9–11^. Τhey are implicated in many diseases, and there is growing evidence that they could be used as biomarkers^12,13^.

This review begins with an overview of the biogenesis, detection, and function of circRNAs, followed by a discussion of the current state of circRNA research in PD and associated processes, and concludes with circRNA-based biomarker studies in PD.

## Biogenesis, expression, and degradation

**Fig. 1:**
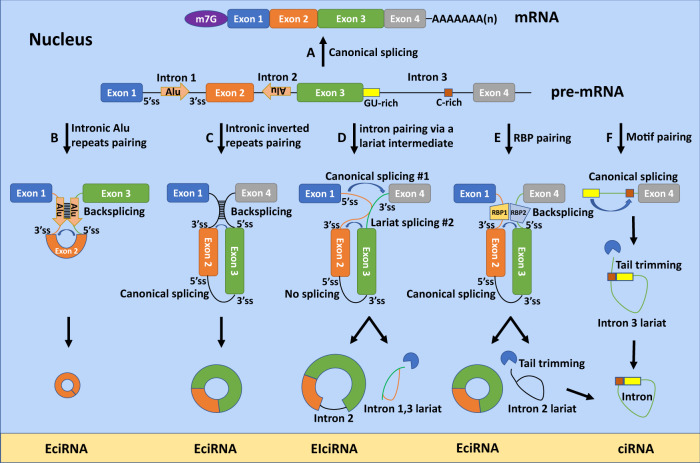
Biogenesis of circRNA types. Canonical splicing generates a mature mRNA from a pre-mRNA (**A**). CircRNAs are generated from exons, introns, or both exons and introns. Exonic circRNAs (EciRNAs) are formed when flanking introns bridged by the pairing of reverse complementary sequences or by RNA binding proteins undergo backsplicing (5′ splice site is covalently linked to 3′ splice site) (**B**) or backsplicing in combination with canonical splicing (**C**, **E**). Exo-intronic circRNAs (EIciRNAs) are formed when introns are retained (**D**). Intronic circRNAs (ciRNAs) are derived from an intron that is excised from a pre-mRNA by canonical splicing when a 7-nt GU rich element at the 5′ss and an 11-nt C-rich element near the branchpoint site pair to form a lariat intron that evades canonical debranching and exonucleolytic degradation. The 3′ tail downstream from the branchpoint is then trimmed, resulting in a circular RNA (**F**).

Most circRNAs are generated from pre-mRNAs, yet some are formed from other RNA species such as pre-tRNAs^14^. According to exon-intron content, we distinguish three subtypes: exonic, exo-intronic, and intronic circRNAs (Fig. 1). Exonic circRNAs are the most abundant of the three and localize in the cytoplasm, where they are exported from the nucleus in a length-dependent manner^15^. They originate from a single or, more commonly, multiple exons. A minimal length of about 300 nucleotides for single exons is typically required for backsplicing^16^. Exo-intronic circRNAs contain both exons and introns, while intronic circRNAs contain only introns; both of these later types are predominantly found in the nucleus^17,18^.

Both *cis* and *trans* factors determine successful backsplicing by the spliceosomal machinery^19^. *Cis* factors are inverted repeats in introns that bring the splice sites into physical proximity promoting circularization. It is estimated that 90% of human circRNAs have complementary Alu repeat elements in their flanking introns^20^. *Trans* factors are RNA binding proteins (RBPs) that regulate circularization by either bridging the flanking introns (e.g., protein quaking [QKI]^21^, fused in sarcoma [FUS]^22^, heterogeneous nuclear ribonucleoprotein L [HNRNPL]^23^, neuro-oncological ventral antigen 2 [NOVA2]^24^), stabilizing RNA duplex formation at the back-spliced junction (e.g., nuclear factor 90/110 [NF90/110]^25^), destabilizing duplex formation (e.g., DExH-box helicase 9 [DHX9]^26^) or inhibiting duplex formation by RNA-editing (adenosine deaminase RNA specific 1 [ADAR1]^20^). The levels of spliceosome also regulate circRNA biogenesis; depletion of spliceosomal components or treatment with splicing inhibitors favors the production of circRNAs over linear mRNAs, perhaps due to a more efficient assembly of exon definition complexes across single or fewer exons^27,28^. Interestingly, the co-transcriptional biogenesis of circRNAs has also been shown to reduce linear host mRNA levels and change downstream splice-site choice in some mRNAs^29,30^.

CircRNAs are widely conserved (28% from mouse to human) and more abundant in the brain than most other tissue^11,31,32^. Over 100,000 circRNAs are expressed in the human brain with a median of three circRNAs per gene, derived primarily from distinct back-spliced junctions within genes^11,32–34^. Many circRNAs are organ-specific, along with their host genes, which are enriched with tissue-specific biological functions^9^. In the brain, circRNA host gene functions are enriched in neurotransmitter secretion, synaptic activities, and neuron maturation^9,11,31,35^. Notably, they are regulated independently from their linear counterparts^11,31^, with some 60% of central nervous system circRNAs being upregulated during development, especially throughout synaptogenesis, while only 2% of their linear isoforms display this tendency^9^. CircRNAs also accumulate during aging, with this trend appearing to be characteristic for brain tissue^36^.

The mechanisms by which circRNAs are degraded are not well-understood. CircRNAs do not have free 5′ and 3′ ends, hence, their decay should be mediated by endoribonucleolytic cleavage. Four alternative mechanisms have been described so far. One is Argonaute 2 (AGO2)-mediated cleavage that depends on microRNA (miRNA) binding to a near-perfect target site on the circRNA^37^. Another mechanism involves circRNAs that undergo adenosine methylation (N^6^-methyladenosine, m^6^A); in this case, m^6^A attracts the adapter heat-responsive protein 12 (HRSP12) and reader YTH N6-methyladenosine RBP 2 (YTHDF2) proteins on circRNA that recruit endoribonuclease RNase P/MRP to initiate circRNA degradation^38^. A third mechanism implicates the primary sequence of the circRNAs following specific cell stimulation (such as viral infection); here, circRNAs that form 16–26 bp imperfect RNA duplexes serve as substrates for endoribonuclease RNase L-mediated degradation^39^. Lastly, binding of the up-frameshift suppressor 1 homolog (UPF1) and endoribonuclease Ras GTPase-activating protein-binding protein 1 (G3BP1) in highly-structured circRNAs, under normal but not stress conditions, triggers unwinding and cleavage of these circRNAs in an as yet poorly defined process^40^.

## Detection

Genome-wide detection of circRNAs depends on identifying RNA-sequencing (RNA-seq) short-reads that are uniquely mapped to back-spliced junctions. For this purpose, several qualitative/quantitative tools have been developed^41–43^. In addition, to experimentally determine the full-length sequence and exact exonic composition of circRNAs, rolling circle amplification followed by nanopore long-read sequencing has also been used^32^. Further, the specificity of circRNA detection can be increased by ribosomal depletion or RNase R treatment to digest linear RNAs;^4^ however, aggressive RNase R treatment may also degrade circRNAs^44^ and eliminate the possibility of obtaining the ratio of circRNA to linear transcripts. Microarray, utilizing probes targeting the back-splice site of each circRNA, is another widely employed method for circRNA profiling and maybe more efficient than RNA-seq when it comes to low abundant circRNAs^45^. RT-PCR is a rapid and sensitive method used to detect circRNAs by utilizing primers that span the back-spliced junction^46^. Northern blot analysis is a reliable but cumbersome biochemical method to detect and validate circRNA species directly. Probes are designed to either detect splice junctions or bind to linear and circRNA, distinguished by electrophoresis since circRNAs migrate slower in a polyacrylamide gel^44,47^. Lastly, a technique used to detect the subcellular localization of circRNAs is RNA fluorescence in situ hybridization, in which fluorescent probes spanning the junction site are applied to fixed cells^48^.

## Function

**Fig. 2:**
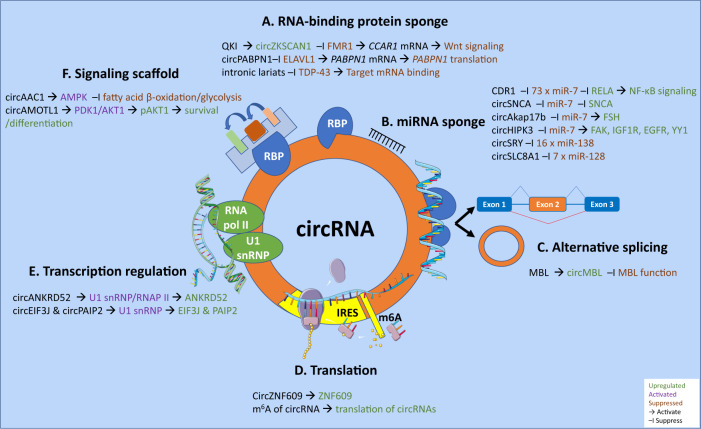
Regulatory functions of circRNAs. CircRNAs function as sponges for RNA binding proteins (**A**) and miRNAs (**B**) to regulate mRNA expression. They also compete with canonical pre-mRNA splicing to induce alternative splicing (**C**). Harboring an IRES motif or by enhanced N^6^ methylation of adenosine residues, they are translated into proteins or peptides (**D**). They directly interact with RNA pol II and U1 snRNP to regulate transcription (**E**), and they function as protein scaffolds for intracellular signaling (**F**). Some elements in this image were obtained from Servier Medical Art (http://smart.servier.com/), permissible to use under a Creative Commons Attribution 3.0 Unported License.

CircRNAs regulate gene expression at multiple levels in both the nucleus and cytosol. They induce transcription and alternative splicing, neutralize miRNAs and RBPs, act as signaling scaffolds, and are translated into functional proteins (Fig. 2).

### Regulation of transcription and splicing

In the nucleus, intronic and exo-intronic circRNAs regulate transcription by interacting with U1 small nuclear ribonucleoprotein (U1 snRNP) and RNA II polymerase. For instance, intronic circANKRD52 (derived from the second intron of ankyrin repeat domain 52 [ANKRD52] gene) directly interacts with elongation pol II machinery to promote transcription of its parental gene^18^. Similarly, exo-intronic circEIF3J and circPAIP2 bind U1 snRNP and likely interact with RNA pol II to also enhance transcription of parental eukaryotic translation initiation factor 3 subunit J (EIF3J) and polyA-binding protein-interacting protein 2 (PAIP2) genes, respectively^17^.

circMBL (derived from muscleblind [MBL/MBNL1] gene) appears to autoregulate its expression and titrate splicing factor MBL/MBNL1 levels in cells to modulate splicing; when MBL levels are high, MBL binds to conserved MBL binding sites in the flanking introns of the circRNA in the pre-mRNA of MBL forcing it to back-splice and form circMBL^29^. High levels of circMBL bind MBL protein, blocking circMBL production and allowing the synthesis of mature *MBL* mRNA^29^.

### miRNA and protein decoys

Several exonic circRNAs function as sponges for miRNAs and RBPs inhibiting their interaction with mRNA targets in the cytoplasm. Perhaps, the best-known example of a miRNA decoy is cerebellar degeneration-related protein 1 antisense (CDR1as, also known as ciRS-7), a conserved and highly abundant circRNA in the mammalian brain. This circRNA contains 73 binding sites for miR-7 (among other miRNA-binding sites) and, therefore, acts as an efficient sponge for it^4,49^. Importantly, critical proteins implicated in neurodegeneration processes, including the ubiquitin-protein ligase A (UBE2A), which catalyzes the proteolytic clearing of toxic amyloid peptides in AD, and alpha-synuclein (SNCA), which accumulates in PD/AD, are known targets of miR-7^50,51^. CDR1as is highly expressed in excitatory neurons, and mice lacking CDR1as exhibit neuropsychiatric disorders with dysfunctional synaptic transmission^52^. In addition, CDR1as is downregulated in the brain of patients with AD^53^. Another circRNA that appears to have significant sponging activity is the testis-specific circSRY which contains 16 target sites for miR-138^49^ and circSLC8A1 that harbors seven binding sites for miR-128, an abundant and brain-restricted miRNA that governs neuronal excitability and motor behavior^54–57^. However, most circRNAs do not contain multiple miRNA-binding sites, and exons that form circRNAs do not exhibit greater cluster densities for AGO2 than neighboring linear exons^58^. Furthermore, to act as efficient miRNA sponges, the circRNAs would need to be expressed at consequential levels within the cell, which is not the case for most circRNAs^7,58^. Nevertheless, numerous circRNAs with a single miRNA-binding site have been shown to efficiently sponge miRNAs and achieve a measurable effect^59–61^.

Several reports have revealed that circRNAs efficiently sponge RBPs and other proteins. circZKSCAN1 was shown to sequester fragile X mental retardation protein 1 (FMR1, also known as FMRP), preventing it from binding to and promoting β-catenin-binding protein-cell cycle and apoptosis regulator 1 (*CCAR1*) mRNA translation, and thereby decreasing Wnt signaling^62^. Similarly, high levels of circPABPN1 prevent human antigen R (HuR, also known as ELAVL1) from binding to its cognate linear polyA-binding protein nuclear 1 (*PABPN1*) mRNA, resulting in reduced PABPN1 translation^63^. In a screen for modifiers of TAR DNA-binding protein 43 (TDP-43, also known as TARDBP) toxicity, knockdown of debranching RNA lariats 1 (DBR1), an enzyme that cleaves the 2′–5′ phosphodiester linkage at the branchpoint of lariat intron pre-mRNAs after splicing and converts them into linear molecules that are subsequently degraded, displayed potent suppressor activity. Authors found that intronic lariat species that accumulated in the cytoplasm of dbr1Δ yeast cells acted as decoys for cytoplasmic TDP-43, preventing it from interfering with essential cellular RNA targets and other RBPs^64^.

### Protein scaffolds

CircRNAs have also been recognized as protein scaffolds. In one such case, circACC1 functions to stabilize and promote the enzymatic activity of AMP-activated protein kinase (AMPK) holoenzyme by forming a ternary complex with the regulatory β and γ subunits. In response to serum deprivation, circACC1 is preferentially produced over acetyl-CoA carboxylase alpha (ACC1, also known as ACACA), an enzyme that catalyzes the first and rate-limiting step of *de novo* fatty acid biosynthesis. Hence, circACC1 increased levels serve as a sensitive indicator and economical platform to elicit AMPK activation for the modulation of fatty acid β-oxidation and glycolysis in response to metabolic stress^65^.

Another example of a scaffolding circRNA is circAMOTL1, which binds to pyruvate dehydrogenase kinase 1 (PDK1) and AKT serine/threonine kinase 1 (AKT1), leading to AKT1 phosphorylation at T308 by PDK1 and nuclear translocation, where it exerts antiapoptotic and differentiation functions^66^.

### Translation

CircRNAs are not necessarily noncoding as most of them are produced from coding segments and reside in the cytoplasm. Having a circular form prevents them from receiving a 7-methylguanosine cap and a polyA tail that supports high levels of translation. However, several possess an internal ribosome entry site (IRES) that can support translation initiation, independent of the 5′ cap structure^67^. Such an example is circZNF609 that contains a 753-nucleotide open reading frame spanning from the start codon, in common with the linear transcript, and terminating at an in-frame stop codon created upon circularization. CircZNF609 is associated with heavy polysomes, and it is translated into a protein in a cap-independent manner by using an IRES sequence at the conserved 5′UTR^68^.

In addition, adenosine methylation (m^6^A) in RNAs, the most abundant base modification of RNA, is cell-type specific and widespread on circRNAs derived from exons not methylated in pre-mRNAs^69^. Importantly, this modification promotes efficient initiation of peptide/protein translation in a cap-independent manner not only for particular mRNAs^70^ but also circRNAs with hundreds of circRNAs displaying this translational potential, particularly during stress^71^. Interestingly, paraquat, a common herbicide and oxidative stress inducer, drives a distinct m^6^A modification pattern of circRNAs^72^.

## CircRNAs in PD pathogenetic processes

**Fig. 3:**
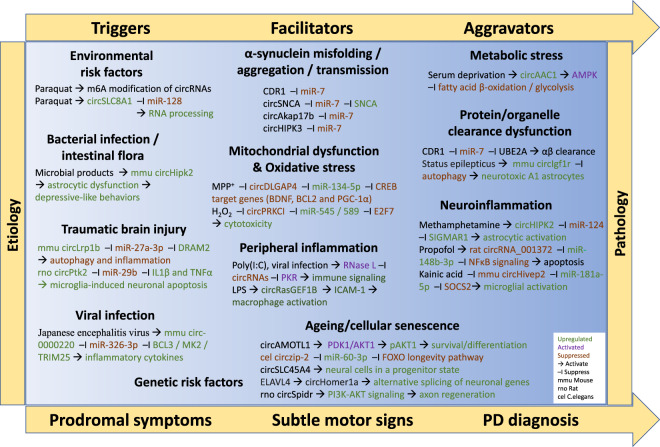
CircRNAs are closely associated with processes implicated in PD pathogenesis. Multiple mechanisms engage their differential expression in neurodegenerative pathways, including inflammation, oxidative stress, cellular senescence, and regulation of SNCA expression.

Given the importance of alternative splicing in generating transcriptome diversity that supports a wide range of biological pathways, particularly in the brain, it is likely that circRNAs will play a substantial role in the pathogenesis of PD. Hereby, insights into circRNA deregulation in PD and the role of circRNAs in PD-associated processes are described in detail (Fig. 3).

### Differential expression

Two studies have identified differentially expressed (DE) circRNAs in different brain regions of patients with PD and the MPTP-induced mouse PD model. In the first study, circRNA levels in 27 control and 42 PD tissue samples from SN, medial temporal gyrus, and amygdala were measured by RNA-seq^73^. Following correction for the cell composition, authors found that in the healthy SN, circRNAs accumulate in an age-dependent manner, while in the SN of patients with PD, this correlation is lost and the number of circRNAs is reduced. Interestingly, the levels of circRNAs were increased in the other two regions of PD patients. Twenty-four circRNAs were DE in all three tissues. These were circNTRK2, circSLC8A1, circRHBDD1, circAFF4, circTACC1, circSTIL, circZHX3, circZGFR1, circNTRK3, circRUFY2, circSLC2A13, circNEBL, circADGRB3, circTAMM41, circ-0139117, circAKT3, circ-0006005, circKDM4C, circARL6IP1, circLRBA, circRER, circNEBL, and circINTS6L^73^.

In the second study, C57BL/6J mice were intraperitoneally injected with 1-methyl-4-phenyl-1,2,3,6-tetrahydropyridine (MPTP) solution at a dose of 20 mg/kg, every day for 10 days. Ten days later, RNA-seq analysis was performed in tissues from different brain regions (3 control and 3 MPTP treated mice were used)^74^. They identified 24, 66, 71, and 121 DE circRNAs in the cerebral cortex, hippocampus, striatum, and cerebellum, respectively. The Kyoto encyclopedia of genes and genomes (KEGG) pathway analysis of the DE circRNA parental genes revealed differences depending on the type of brain tissue. AD and calcium signaling were enriched in the hippocampus, whereas autophagy and mitogen-activated protein kinase (MAPK) signaling in the striatum. Further, PD, axon guidance, and cGMP-dependent protein kinase G signaling were enriched in the cerebellum, while no pathway was enriched in the cerebral cortex^74^.

### Aging

Aging is the most significant single independent risk factor for the development of PD. A combination of foreseeable and random processes leads to the accumulation of unrepaired cellular damage, weakening cellular processes and straining compensatory mechanisms. It is thought that lifestyle and environmental factors account for most human differences in aging, with genes responsible for perhaps only 25% of variance^75^.

Two studies, so far, have examined the effects of aging on circRNA expression. In the first study, after probing circRNAs that were DE between 10 and 20 years old in rhesus macaque brain samples, 3% of circRNAs (475 out of 17,050, *p* value < 0.05) were found to be associated with brain aging (or 73 circRNAs with *p* value < 0.01)^76^. Further investigation of eleven circRNAs derived from receptor, protein kinase, and calcium signaling parental genes (circCACNA1E, circMAP2K5, circERBB4, circLATS1, circGABBR2, circBMPR2, circGRIA1, circCACNA2D1, circMASTL, circMAPK1, and circCACNB2) revealed that nine circRNAs were negatively correlated while two circRNAs positively correlated with parental mRNA expression^76^, indicating that circRNA expression interferes with mRNA/protein output. To back up their results, the researchers used siRNA to knockdown the expression of two circRNAs that decreased with aging, circCACNA2D1 and circCACNA1E, in cultures of hippocampal neurons and confirmed that host mRNAs were upregulated^76^.

In the second study, the differential expression profile of circRNAs in the hippocampus of 10-month-old senescence-accelerated mouse prone 8 (SAMP8) mice versus control mice revealed 45 DE circRNAs. In addition, 119 circRNAs exhibited differential expression in 10-month-old SAMP8 versus 5-month-old SAMP8^77^. Validation of six circRNAs by PCR revealed that circ-017963 exhibited the highest decrease in expression during aging. Bioinformatic analysis of its miRNA-mRNA target network revealed that it is enriched for autophagosome assembly, exocytosis, apoptotic process, transport, and RNA splicing^77^.

### SNCA and miR-7

SNCA expression is integral to the pathological process in PD. Point mutations, gene duplication, and triplication events in the SNCA locus have been identified in several families with autosomal dominant early-onset PD^78–80^. Moreover, SNCA is a major component of Lewy bodies (LB) in sporadic PD and dementia with LB, and also of inclusions found in both glial and neuronal cells in multiple system atrophy (MSA)^81^. In vitro cell culture studies indicate that the expression of mutant SNCA can sensitize neurons to toxic challenges, and viral-mediated overexpression of wild-type or mutant SNCA within nigral neurons of rodents and non-human primates has led to progressive motor dysfunction mimicking motor symptoms in PD patients^82–84^. SNCA is a target of miR-7^50,85^, and its accumulation, as well as aggregation in vitro and in vivo, is linked to miR-7 levels in neurons^86^. Further, miR-7 levels decrease in the SN of patients with PD, and depletion of miR-7 levels using a miR-decoy produces a loss of nigral dopaminergic neurons, accompanied by a reduction of striatal dopamine content^86^.

As described in miRNA and protein decoys section, miR-7 levels are physiologically regulated by the highly expressed and brain-enriched CDR1as^4,49^. Interestingly, CDR1as knockout mice display decreased levels of miR-7, indicating that this circRNA may not only sponge miR-7 function but also enhance miR-7 stability or intracellular transport and localization^52^.

Additional circRNAs that sequester miR-7 have been identified. miR-7 is highly expressed in the pituitary gland^87^, and circAkap17b, a pituitary-specific circRNA, was shown to sponge miR-7 de-repressing the expression and secretion of its target follicle-stimulating hormone^60,87^. Further, by using a biotinylated circHIPK3 probe to perform RNA pull-down assay, miR-7 was the only miRNA that was copiously pulled-down by circHIPK3, a finding that was subsequently verified by additional assays^88^. Moreover, ectopic expression of circHIPK3 effectively reversed miR-7 inhibition of its targets in cancer cells^88^. Since circHIPK3 is highly abundant in the brain^11^, its potential role in regulating SNCA expression is worth being evaluated in a neuronal context.

The gene encoding for *SNCA* mRNA can also produce circRNAs. This circRNA is derived from the proximal 3′UTR of *SNCA* mRNA that contains the high-affinity site for miR-7. It was shown that circSNCA could efficiently sponge miR-7, *in culture*^89^. Further, pramipexole, a dopamine agonist, was found to decrease circSNCA levels leading to increased miR-7 and decreased SNCA protein levels in SH-SH5Y cells. In addition, higher circSNCA expression was associated with increased expression of pro-apoptotic proteins and decreasing levels of autophagy-associated protein LC3B-II^89^.

In another study, circzip-2 (cel-circ-000006) was identified as one of the two most highly expressed circRNAs in *C. elegans*^4,90^. Interestingly, circzip-2 levels were downregulated by 18-fold in the SNCA overexpression PD model compared to the wild-type strain of *C. elegans*. Further analysis revealed that circzip-2 potentially serves as a sponge for miR-60-3p that represses mRNAs of the forkhead box O (FOXO) pathway, which in turn has a protective role against the development of PD and aging^90^.

Finally, in MSA, RNA-seq analysis on six control and six subjects with MSA revealed five circRNAs, namely, circIQCK, circMAP4K3, circEFCAB11, circDTNA, and circMCTP1, as specifically over-expressed in MSA frontal cortex. The DE levels of these five circRNAs, whose host mRNAs expression were not altered, turned out to originate from the white matter^91^.

### Neuroinflammation

One of the hallmarks of PD pathophysiology is chronic inflammation. In patients with PD, autopsies of the post-mortem human brain have revealed the role of both innate and adaptive immunity. T lymphocytes, activated microglia, and astrocytes have been found in the SN and other affected areas in PD patients, along with an increase in the expression of proinflammatory mediators^92^. Preclinical studies in animals utilizing either PD toxins or SNCA overexpression have also revealed neuroinflammation as a critical contributor to the pathology, and although it may not be the primary cause in these cases, it is involved in self-perpetuating deleterious events that lead to protracted neuronal degeneration. Furthermore, recent studies concerning, for instance, the gut microbiota composition, have emphasized the ever-increasing role of peripheral inflammation in PD pathogenesis^92^. With respect to the circRNA transcriptomes of astrocytes and microglia, it was revealed that they are unique from one another and that the most abundant circRNAs are expressed by parent genes co-expressing linear RNAs in low abundance, indicating critical roles of circRNAs for glial function^93^.

Several circRNAs have been implicated in neuroinflammation but not in the precise context of PD as yet. In traumatic brain injury (TBI) caused by controlled cortical impact, an animal model with widespread inflammation, high-throughput RNA-seq (HTS) in RNA extracted from mouse cortex revealed 191 DE circRNAs. Enrichment analyses indicated that inflammation, cell death, and damage repair were the main biological processes related to DE circRNA host genes^94^. In the same animal model, circ-010705 (derived from LDL receptor-related protein 1B [Lrp1b] gene) was found to be upregulated, while its target miR-27a-3p downregulated. Accordingly, the mRNA and protein expression of DNA damage regulated autophagy modulator 2 (DRAM2), the target of miR-27a-3p, was elevated in TBI. Consequently, knockdown of circLrp1b or miR-27a-3p overexpression, in vivo, suppressed TBI-induced autophagy and inflammation, while DRAM2 restoration abolished this effect^95^.

Similarly, in cerebral ischemia, microglia become activated, inducing inflammatory responses. Performing oxygen-glucose deprivation (OGD) in cultures of microglial cells, it was shown that circPtk2, IL-1β, and TNFα levels were increased with a concomitant decrease in the levels of miR-29b^96^, a microRNA that is also DE in PD^97^. Conditioned media from these microglial cultures promoted hippocampal neuron apoptosis, which was reversed by prior miR-29b overexpression in microglia. Subsequently, it was shown that OGD-activated microglia-induced neuronal apoptosis is mediated by circPTK2 that sequesters miR-29b in microglia^96^. In the propofol-induced neurotoxicity and neuroinflammation rat model, levels of circ-001372 decrease while levels of IL-1β, IL-6, IL17, and IL-18 cytokines increase, resulting in hippocampal neuronal apoptosis^98^. Overexpression of circ-001372 rescued PC12 cells from apoptosis by sponging miR-148b-3p, an effect that was accompanied by enhanced PI3K/AKT and decreased NFκB signaling^98^.

In Japanese encephalitis, characterized by the uncontrolled release of inflammatory cytokines in the brain, HTS identified 180 DE circRNAs in JEV-infected murine brains. Gene ontology and KEGG enrichment analyses revealed that the circRNA parental genes were related to neurotransmission, histone modifications, transcription misregulation, and inflammation-associated calcium signaling^99^. Additional analysis of one of the DE circRNAs, circ-0000220, revealed that either knockdown of this circRNA or overexpression of its target miR-326-3p in BV-2 microglia cells lowered the production of inflammatory cytokines^99^.

Several studies have also looked into the astrocytic activation by circRNAs^59,98^. Methamphetamine-induced degeneration of dopaminergic neurons in the SNpc is associated with extensive reactive astrogliosis in the striatum^59,100–102^. Sigma non-opioid intracellular receptor 1 (SIGMAR1, also known as OPSR1) plays a crucial role in astrocytic activation, and both lipopolysaccharide and methamphetamine have been shown to induce its expression^59^. Interestingly, SIGMAR1 is a target of miR-124, and miR-124 overexpression can inhibit astrocytic activation via SIGMAR1 both in vitro and in vivo^59^. Further, circHIPK2 acts as an endogenous sponge for miR-124, and treatment with either siRNA or lentiviral shRNA for circHIPK2 significantly inhibits methamphetamine-induced astrocytic activation by lowering the expression of SIGMAR1 in vitro and in vivo^59^.

Taken together, it can be inferred from these data that circRNAs are indispensable for microglial and astrocytic activation, and forthcoming studies will establish if these circRNAs are significantly impacting PD pathogenesis.

### Oxidative stress

Oxidative stress plays a crucial role in the cascade leading to dopamine cell degeneration in familial and sporadic PD. Since reactive oxygen species (ROS) are a by-product of oxidative phosphorylation, mitochondria are the primary source of ROS in cells. ROS are formed continuously by all cells in the body, but oxidative stress arises only when there is an imbalance between ROS production and total antioxidant activity^103^. Environmental factors such as neurotoxins and pesticides, dopamine itself, calcium, iron, obesity, inflammation, aging, and mutations are thought to tip the scales in favor of ROS formation and mitochondrial dysfunction^103^.

Nuclear factor, erythroid 2 like 2 (NRF2) is a transcription factor that regulates the cellular redox status. Its activity rises in response to redox perturbation, inflammation, growth factor stimulation, and energy flux, allowing it to orchestrate adaptive responses to various stressors^104^. Microarray analysis of SN and striatum from Nrf2 knockout and wild-type mice revealed 65 and 150 DE circRNAs, respectively^105^. Seventeen DE circRNA were shared between these tissues. circRNA-miRNA-mRNA interaction network analysis revealed that circ-34132 (derived from D430041D05Rik gene), circ-017077 (derived from MAX gene-associated protein [MGA] gene), and circ-015216 (derived from YES proto-oncogene 1 [YES1] gene) are potentially involved in Nrf2-mediated neuroprotection against oxidative stress^105^.

Another HTS analysis of circRNAs in the brain found 3407 different circRNAs expressed only in PD, whereas 1,028 emerged as unique to healthy controls, suggesting disease-related changes in the backsplicing process^73^. Among the DE circRNAs in the SN of individuals with PD was circSLC8A1, whose expression was increased^73^. circSLC8A1 levels were also increased in SH-SY5Y cells exposed to the oxidative stress-inducing agent paraquat but were decreased by simvastatin, a cholesterol-reducing drug, and leucine-rich repeat kinase 2 inhibitor PF-06447475^73^. Enrichment analysis of terms for the DE genes regulated by circSLC8A1 knockdown included RNA binding, nucleoplasm, and SWI/SNF superfamily complex^73^.

In the MPTP-induced mouse model of PD and MPP^+^-treated cell lines, circDLGAP4 expression was found reduced^106^. circDLGAP4 silencing using siRNAs, *in culture*, led to mitochondrial damage, decreased autophagy, and enhanced apoptosis. In contrast, overexpression of circDLGAP4 attenuated the effects of MPP^+^ in SH-SY5Y and MN9D cells. Further research suggested that circDLGAP4 exerted its functions by sponging miR-134-5p and promoting the expression of cAMP-responsive element binding protein 1 target genes, including brain-derived neurotrophic factor, B-cell lymphoma 2, and peroxisome proliferator-activated receptor-γ coactivator 1α^106^. Another study corroborated these effects in which overexpression of circDLGAP4 attenuated neurological deficits and decreased infarct area and blood-brain barrier damage in the transient middle cerebral artery occlusion mouse stroke model^107^.

Lastly, in cultures of SH-SY5Y cells, hydrogen peroxide (H_2_O_2_) was shown to downregulate circPRKCI expression in a dose-dependent manner, while circPRKCI’s two targets miR-545 and miR-589 were accumulated with a concomitant reduction of their mRNA target, the transcription factor E2F transcription factor 7 (E2F7)^108^. Ectopic overexpression of circPRKCI or application of miR-545/589 antagomiRs in either SH-SY5Y cells or primary neurons attenuated H_2_O_2_-induced cytotoxicity, indicating that targeting this novel cascade may protect neurons from oxidative stress^108^.

## The biomarker potential of circRNAs

PD is currently diagnosed using clinical criteria and neuroimaging and is monitored by rating scales related to motor and non-motor features^109^. Rating scales are often subjective and influenced by periodic fluctuations in symptoms and effective symptomatic therapies, while neuroimaging techniques, such as dopamine transporter-single-photon emission computed tomography, provide a quantifiable measure of disease progression but are limited in terms of practicality and costs^110^.

CircRNAs are promising biomarkers since they are highly stable, diverse, and abundant in the brain, display cell- and tissue-specific expression, do not get modified like proteins, hence levels directly correlate with activity, and can be accurately quantified by routine and fast laboratory methods, such as RT-PCR. Further, circRNAs have been identified in various noninvasive biofluids, including peripheral blood mononuclear cells (PBMCs), saliva, and plasma, and have been put forward as biomarkers for AD, PD, schizophrenia, and bipolar disorder^46,111–114^. Notably, a recent study using brain tissue samples from patients with AD identified a significant association between circRNA expression and diagnosis, clinical dementia severity, and neuropathological severity. In addition, pre-symptomatic changes in circRNA expression were also identified, exemplifying their great potential to serve as biomarkers for AD and other neurodegenerative diseases^115^.

Three studies, so far, have identified DE circRNAs in the peripheral blood of patients with PD. The first study used 60 paired PBMCs from idiopathic PD patients and healthy controls in RT-qPCR analysis^46^. Out of the 48 circRNAs ultimately examined, six circRNAs (circSLAIN1, circDOP1B, circRESP1, circMAPK9, circPSEN1, circHOMER1) were significantly downregulated in patients with PD. The classifier that best-distinguished PD consisted of four circRNAs with an area under the curve of 0.84. Interestingly, investigating cross‐linking immunoprecipitation‐sequencing data, the authors found that the RNA‐binding proteins bound by most of these deregulated circRNAs included the neurodegeneration‐associated proteins FUS, TDP-43, FMR1, and ataxin 2 (ATXN2) implicating them, along with few other studies until now, in PD pathogenesis^46^. The second study used 4 paired total blood from PD patients and controls in RNA-seq analysis^116^. There were 129 upregulated and 282 downregulated circRNAs in patients with PD. The top ten deregulated circRNAs included circHBB, circSIN3A, circITGAL, circFAM13B, circFBXW7, circRBM39, circSLTM, circYY1AP1, circPCMTD1, and circRBM33^116^. The third study used three paired plasma from PD patients (stage 1 to stage 4–5) and controls in microarray analysis^117^. Six circRNAs, namely circFAM83H, circMRPL53, circRPTOR, circARID1B, circTCONS-l2-00002816, and circHUWE1 were upregulated in all three patients with PD. Among these circRNAs, circFAM83H, circARID1B, circTCONS-l2-00002816, and circHUWE1 were presented with rapidly increased levels as the disease progressed. Following that, a two-step RT-qPCR validation screening with additional samples revealed that circARID1B and circTCONS-l2-00002816 could predict early-stage PD, while circFAM83H, circARID1B, circTCONS-l2-00002816, and circHUWE1 could discriminate late-stage PD from early-stage PD^117^.

## Conclusions

The discovery and analysis of circRNAs have once again exposed the complexity of eukaryotic transcriptomes and the vast array of biological functions that set them apart from other types of RNAs. HTS studies have identified a great number of circRNAs that are DE in PD-related processes. Research efforts need to be channeled toward characterizing their specific regulatory roles and evaluating the extent to which they contribute to PD pathology. Currently, available biomarkers for PD are not sensitive or specific enough. Properties such as stability, diversity, tissue specificity, and expression in noninvasive biofluids mark circRNAs as potential and promising biomarkers for PD’s clinical diagnosis and prognosis. Finally, more research into their interplay with other regulatory networks involving ncRNAs and proteins will aid in the understanding of PD pathogenesis and provide a valuable resource for the development of new therapeutic and diagnostic regimes.

## References

[1] Sveinbjornsdottir S (2016). The clinical symptoms of Parkinson’s disease. J. Neurochem..

[2] Dickson DW (2018). Neuropathology of Parkinson’s disease. Parkinsonism Relat. Disord..

[3] Klein C, Schlossmacher MG (2007). Parkinson disease, 10 years after its genetic revolution: multiple clues to a complex disorder. Neurology.

[4] Memczak S (2013). Circular RNAs are a large class of animal RNAs with regulatory potency. Nature.

[5] Salzman J, Gawad C, Wang PL, Lacayo N, Brown PO (2012). Circular RNAs are the predominant transcript isoform from hundreds of human genes in diverse cell types. PLoS ONE.

[6] Zaphiropoulos PG (1996). Circular RNAs from transcripts of the rat cytochrome P450 2C24 gene: correlation with exon skipping. Proc. Natl Acad. Sci. USA.

[7] Jeck WR (2013). Circular RNAs are abundant, conserved, and associated with ALU repeats. RNA.

[8] Schwanhausser B (2011). Global quantification of mammalian gene expression control. Nature.

[9] Mahmoudi E, Cairns MJ (2019). Circular RNAs are temporospatially regulated throughout development and ageing in the rat. Sci. Rep..

[10] Veno MT (2015). Spatio-temporal regulation of circular RNA expression during porcine embryonic brain development. Genome Biol..

[11] Rybak-Wolf A (2015). Circular RNAs in the mammalian brain are highly abundant, conserved, and dynamically expressed. Mol. cell.

[12] Rophina, M., Sharma, D., Poojary, M. & Scaria, V. Circad: a comprehensive manually curated resource of circular RNA associated with diseases. *Database* **2020**10.1093/database/baaa019 (2020). 10.1093/database/baaa019 PMC7100626 32219412

[13] Yao D (2018). Circ2Disease: a manually curated database of experimentally validated circRNAs in human disease. Sci. Rep..

[14] Lu Z (2015). Metazoan tRNA introns generate stable circular RNAs in vivo. RNA.

[15] Huang C, Liang D, Tatomer DC, Wilusz JE (2018). A length-dependent evolutionarily conserved pathway controls nuclear export of circular RNAs. Genes Dev..

[16] Kramer MC (2015). Combinatorial control of Drosophila circular RNA expression by intronic repeats, hnRNPs, and SR proteins. Genes Dev..

[17] Li Z (2015). Exon-intron circular RNAs regulate transcription in the nucleus. Nat. Struct. Mol. Biol..

[18] Zhang Y (2013). Circular intronic long noncoding RNAs. Mol. Cell.

[19] Starke S (2015). Exon circularization requires canonical splice signals. Cell Rep..

[20] Ivanov A (2015). Analysis of intron sequences reveals hallmarks of circular RNA biogenesis in animals. Cell Rep..

[21] Conn SJ (2015). The RNA binding protein quaking regulates formation of circRNAs. Cell.

[22] Errichelli L (2017). FUS affects circular RNA expression in murine embryonic stem cell-derived motor neurons. Nat. Commun..

[23] Fei T (2017). Genome-wide CRISPR screen identifies HNRNPL as a prostate cancer dependency regulating RNA splicing. Proc. Natl Acad. Sci. USA.

[24] Knupp D, Cooper DA, Saito Y, Darnell RB, Miura P (2021). NOVA2 regulates neural circRNA biogenesis. Nucleic Acids Res..

[25] Li X (2017). Coordinated circRNA biogenesis and function with NF90/NF110 in viral infection. Mol. Cell.

[26] Aktas T (2017). DHX9 suppresses RNA processing defects originating from the Alu invasion of the human genome. Nature.

[27] Liang D (2017). The output of protein-coding genes shifts to circular RNAs when the pre-mRNA processing machinery is limiting. Mol. Cell.

[28] Wang M, Hou J, Muller-McNicoll M, Chen W, Schuman EM (2019). Long and repeat-rich intronic sequences favor circular RNA formation under conditions of reduced spliceosome activity. iScience.

[29] Ashwal-Fluss R (2014). circRNA biogenesis competes with pre-mRNA splicing. Mol. Cell.

[30] Koh W (2016). Dynamic ASXL1 exon skipping and alternative circular splicing in single human cells. PLoS ONE.

[31] You X (2015). Neural circular RNAs are derived from synaptic genes and regulated by development and plasticity. Nat. Neurosci..

[32] Xin R (2021). isoCirc catalogs full-length circular RNA isoforms in human transcriptomes. Nat. Commun..

[33] Gokool A, Anwar F, Voineagu I (2020). The landscape of circular RNA expression in the human brain. Biol. Psychiatry.

[34] Liu Z (2019). Detection of circular RNA expression and related quantitative trait loci in the human dorsolateral prefrontal cortex. Genome Biol..

[35] Zimmerman, A. J. et al. A psychiatric disease-related circular RNA controls synaptic gene expression and cognition. *Mol. Psychiatry*10.1038/s41380-020-0653-4 (2020). 10.1038/s41380-020-0653-4 PMC7577899 31988434

[36] Gruner H, Cortes-Lopez M, Cooper DA, Bauer M, Miura P (2016). CircRNA accumulation in the aging mouse brain. Sci. Rep..

[37] Hansen TB (2011). miRNA-dependent gene silencing involving Ago2-mediated cleavage of a circular antisense RNA. EMBO J..

[38] Park OH (2019). Endoribonucleolytic cleavage of m(6)A-containing RNAs by RNase P/MRP complex. Mol. Cell.

[39] Liu CX (2019). Structure and degradation of circular RNAs regulate PKR activation in innate immunity. Cell.

[40] Fischer JW, Busa VF, Shao Y, Leung AKL (2020). Structure-mediated RNA Decay by UPF1 and G3BP1. Mol. Cell.

[41] Cheng J, Metge F, Dieterich C (2016). Specific identification and quantification of circular RNAs from sequencing data. Bioinformatics.

[42] Gao Y, Wang J, Zhao F (2015). CIRI: an efficient and unbiased algorithm for de novo circular RNA identification. Genome Biol..

[43] Zheng Y, Zhao F (2018). Detection and reconstruction of circular RNAs from transcriptomic data. Methods Mol. Biol..

[44] Zhang Y, Yang L, Chen LL (2016). Characterization of Circular RNAs. Methods Mol. Biol..

[45] Li S (2019). Microarray is an efficient tool for circRNA profiling. Brief. Bioinforma..

[46] Ravanidis, S. et al. Differentially expressed circular RNAs in peripheral blood mononuclear cells of patients with Parkinson’s disease. *Mov. Disorders*10.1002/mds.28467 (2021). 10.1002/mds.28467 PMC8248110 33433033

[47] Schneider T, Schreiner S, Preusser C, Bindereif A, Rossbach O (2018). Northern blot analysis of circular RNAs. Methods Mol. Biol..

[48] Zirkel A, Papantonis A (2018). Detecting circular RNAs by RNA fluorescence in situ hybridization. Methods Mol. Biol..

[49] Hansen TB (2013). Natural RNA circles function as efficient microRNA sponges. Nature.

[50] Doxakis E (2010). Post-transcriptional regulation of alpha-synuclein expression by mir-7 and mir-153. J. Biol. Chem..

[51] Zhao, Y., Alexandrov, P. N., Jaber, V. & Lukiw, W. J. Deficiency in the ubiquitin conjugating enzyme UBE2A in Alzheimer’s disease (AD) is linked to deficits in a natural circular miRNA-7 sponge (circRNA; ciRS-7). *Genes* **7**10.3390/genes7120116 (2016). 10.3390/genes7120116 PMC5192492 27929395

[52] Piwecka, M. et al. Loss of a mammalian circular RNA locus causes miRNA deregulation and affects brain function. *Science* **357**10.1126/science.aam8526 (2017). 10.1126/science.aam8526 28798046

[53] Lukiw WJ (2013). Circular RNA (circRNA) in Alzheimer’s disease (AD). Front. Genet..

[54] Paschou M, Doxakis E (2012). Neurofibromin 1 Is a miRNA target in neurons. PLoS ONE.

[55] Paschou M (2020). Neuronal microRNAs modulate TREK two-pore domain K(+) channel expression and current density. RNA Biol..

[56] Tan CL (2013). MicroRNA-128 governs neuronal excitability and motor behavior in mice. Science.

[57] Zhang, W. et al. MiRNA-128 regulates the proliferation and neurogenesis of neural precursors by targeting PCM1 in the developing cortex. *eLife* **5**10.7554/eLife.11324 (2016). 10.7554/eLife.11324 PMC4769165 26883496

[58] Guo JU, Agarwal V, Guo H, Bartel DP (2014). Expanded identification and characterization of mammalian circular RNAs. Genome Biol..

[59] Huang R (2017). Circular RNA HIPK2 regulates astrocyte activation via cooperation of autophagy and ER stress by targeting MIR124-2HG. Autophagy.

[60] Wang CJ (2020). circAkap17b acts as a miR-7 family molecular sponge to regulate FSH secretion in rat pituitary cells. J. Mol. Endocrinol..

[61] Xiaoying G (2020). CircHivep2 contributes to microglia activation and inflammation via miR-181a-5p/SOCS2 signalling in mice with kainic acid-induced epileptic seizures. J. Cell. Mol. Med..

[62] Zhu YJ (2019). Circular RNAs negatively regulate cancer stem cells by physically binding FMRP against CCAR1 complex in hepatocellular carcinoma. Theranostics.

[63] Abdelmohsen K (2017). Identification of HuR target circular RNAs uncovers suppression of PABPN1 translation by CircPABPN1. RNA Biol..

[64] Armakola M (2012). Inhibition of RNA lariat debranching enzyme suppresses TDP-43 toxicity in ALS disease models. Nat. Genet..

[65] Li Q (2019). CircACC1 regulates assembly and activation of AMPK complex under metabolic stress. Cell Metab..

[66] Zeng Y (2017). A circular RNA binds to and activates AKT phosphorylation and nuclear localization reducing apoptosis and enhancing cardiac repair. Theranostics.

[67] Koukouraki P, Doxakis E (2016). Constitutive translation of human alpha-synuclein is mediated by the 5′-untranslated region. Open Biol..

[68] Legnini I (2017). Circ-ZNF609 is a circular RNA that can be translated and functions in myogenesis. Mol. Cell.

[69] Zhou C (2017). Genome-wide maps of m6A circRNAs identify widespread and cell-type-specific methylation patterns that are distinct from mRNAs. Cell Rep..

[70] Wang X (2015). N(6)-methyladenosine modulates messenger RNA translation efficiency. Cell.

[71] Yang Y (2017). Extensive translation of circular RNAs driven by N(6)-methyladenosine. Cell Res..

[72] Chen N (2021). Paraquat-induced oxidative stress regulates N6-methyladenosine (m(6)A) modification of circular RNAs. Environ. Pollut..

[73] Hanan, M. et al. A Parkinson’s disease CircRNAs Resource reveals a link between circSLC8A1 and oxidative stress. *EMBO Mol. Med.* e11942 10.15252/emmm.201911942 (2020). 10.15252/emmm.201911942 PMC7507321 32715657

[74] Jia, E. et al. Transcriptomic profiling of circular RNA in different brain regions of Parkinson’s disease in a mouse model. *Int. J. Mol. Sci.* **21**10.3390/ijms21083006 (2020). 10.3390/ijms21083006 PMC7216060 32344560

[75] Hindle JV (2010). Ageing, neurodegeneration and Parkinson’s disease. Age Ageing.

[76] Xu K (2018). Annotation and functional clustering of circRNA expression in rhesus macaque brain during aging. Cell Discov..

[77] Huang JL (2018). Comprehensive analysis of differentially expressed profiles of Alzheimer’s disease associated circular RNAs in an Alzheimer’s disease mouse model. Aging.

[78] Chartier-Harlin MC (2004). Alpha-synuclein locus duplication as a cause of familial Parkinson’s disease. Lancet.

[79] Polymeropoulos MH (1997). Mutation in the alpha-synuclein gene identified in families with Parkinson’s disease. Science.

[80] Singleton AB (2003). alpha-Synuclein locus triplication causes Parkinson’s disease. Science.

[81] Spillantini MG, Crowther RA, Jakes R, Hasegawa M, Goedert M (1998). alpha-Synuclein in filamentous inclusions of Lewy bodies from Parkinson’s disease and dementia with lewy bodies. Proc. Natl Acad. Sci. USA.

[82] Kirik D (2002). Parkinson-like neurodegeneration induced by targeted overexpression of alpha-synuclein in the nigrostriatal system. J. Neurosci..

[83] Lo Bianco C, Ridet JL, Schneider BL, Deglon N, Aebischer P (2002). alpha -Synucleinopathy and selective dopaminergic neuron loss in a rat lentiviral-based model of Parkinson’s disease. Proc. Natl Acad. Sci. USA.

[84] Oliveras-Salva M (2013). rAAV2/7 vector-mediated overexpression of alpha-synuclein in mouse substantia nigra induces protein aggregation and progressive dose-dependent neurodegeneration. Mol. Neurodegener..

[85] Junn E (2009). Repression of alpha-synuclein expression and toxicity by microRNA-7. Proc. Natl Acad. Sci. USA.

[86] McMillan KJ (2017). Loss of MicroRNA-7 regulation leads to alpha-synuclein accumulation and dopaminergic neuronal loss in vivo. Mol. Ther..

[87] Wang CJ (2019). Pituitary tissue-specific miR-7a-5p regulates FSH expression in rat anterior adenohypophyseal cells. PeerJ.

[88] Zeng K (2018). CircHIPK3 promotes colorectal cancer growth and metastasis by sponging miR-7. Cell Death Dis..

[89] Sang Q (2018). CircSNCA downregulation by pramipexole treatment mediates cell apoptosis and autophagy in Parkinson’s disease by targeting miR-7. Aging.

[90] Kumar L (2018). Functional characterization of novel circular RNA molecule, circzip-2 and its synthesizing gene zip-2 in C. elegans model of Parkinson’s disease. Mol. Neurobiol..

[91] Chen BJ (2016). Characterization of circular RNAs landscape in multiple system atrophy brain. J. Neurochem..

[92] Troncoso-Escudero P, Parra A, Nassif M, Vidal RL (2018). Outside in: unraveling the role of neuroinflammation in the progression of Parkinson’s disease. Front. Neurol..

[93] Curry-Hyde A (2020). Cell type-specific circular RNA expression in human glial cells. Genomics.

[94] Jiang YJ (2019). Circular ribonucleic acid expression profile in mouse cortex after traumatic brain injury. J. Neurotrauma.

[95] Li H (2020). Dexmedetomidine inhibits inflammatory response and autophagy through the circLrp1b/miR-27a-3p/Dram2 pathway in a rat model of traumatic brain injury. Aging.

[96] Wang H, Li Z, Gao J, Liao Q (2019). Circular RNA circPTK2 regulates oxygen-glucose deprivation-activated microglia-induced hippocampal neuronal apoptosis via miR-29b-SOCS-1-JAK2/STAT3-IL-1beta signaling. Int. J. Biol. Macromol..

[97] Doxakis E (2020). Cell-free microRNAs in Parkinson’s disease: potential biomarkers that provide new insights into disease pathogenesis. Ageing Res. Rev..

[98] Wang, M., Suo, L., Yang, S. & Zhang, W. CircRNA 001372 reduces inflammation in propofol-induced neuroinflammation and neural apoptosis through PIK3CA/Akt/NF-kappaB by miRNA-148b-3p. *J. Invest. Surg.* 1–11 10.1080/08941939.2020.1771639 (2020). 10.1080/08941939.2020.1771639 32506974

[99] Li Y (2020). Genome-wide profiling of host-encoded circular RNAs highlights their potential role during the Japanese encephalitis virus-induced neuroinflammatory response. BMC Genomics.

[100] Granado N (2011). Dopamine D2-receptor knockout mice are protected against dopaminergic neurotoxicity induced by methamphetamine or MDMA. Neurobiol. Dis..

[101] Pu C, Vorhees CV (1993). Developmental dissociation of methamphetamine-induced depletion of dopaminergic terminals and astrocyte reaction in rat striatum. Brain Res. Dev. Brain Res..

[102] Sakoori K, Murphy NP (2010). Reduced degeneration of dopaminergic terminals and accentuated astrocyte activation by high dose methamphetamine administration in nociceptin receptor knock out mice. Neurosci. Lett..

[103] Blesa J, Trigo-Damas I, Quiroga-Varela A, Jackson-Lewis VR (2015). Oxidative stress and Parkinson’s disease. Front. Neuroanat..

[104] Hayes JD, Dinkova-Kostova AT (2014). The Nrf2 regulatory network provides an interface between redox and intermediary metabolism. Trends Biochem. Sci..

[105] Yang JH (2018). The differentially expressed circular RNAs in the substantia nigra and corpus striatum of Nrf2-knockout mice. Cell. Physiol. Biochem..

[106] Feng Z, Zhang L, Wang S, Hong Q (2020). Circular RNA circDLGAP4 exerts neuroprotective effects via modulating miR-134-5p/CREB pathway in Parkinson’s disease. Biochem. Biophys. Res. Commun..

[107] Bai Y (2018). Circular RNA DLGAP4 ameliorates ischemic stroke outcomes by targeting miR-143 to regulate endothelial-mesenchymal transition associated with blood-brain barrier integrity. J. Neurosci..

[108] Cheng Q, Cao X, Xue L, Xia L, Xu Y (2019). CircPRKCI-miR-545/589-E2F7 axis dysregulation mediates hydrogen peroxide-induced neuronal cell injury. Biochem. Biophys. Res. Commun..

[109] Postuma RB (2015). MDS clinical diagnostic criteria for Parkinson’s disease. Mov. Disord..

[110] Bhidayasiri R, Martinez-Martin P (2017). Clinical assessments in Parkinson’s disease: scales and monitoring. Int. Rev. Neurobiol..

[111] Bahn JH (2015). The landscape of microRNA, Piwi-interacting RNA, and circular RNA in human saliva. Clin. Chem..

[112] Li Y (2020). Profiling of differentially expressed circular RNAs in peripheral blood mononuclear cells from Alzheimer’s disease patients. Metab. Brain Dis..

[113] Mahmoudi, E., Green, M. J. & Cairns, M. J. Dysregulation of circRNA expression in the peripheral blood of individuals with schizophrenia and bipolar disorder. *J. Mol. Med.*10.1007/s00109-021-02070-6 (2021). 10.1007/s00109-021-02070-6 33782720

[114] Tan G (2021). The alterations of circular RNA expression in plasma exosomes from patients with schizophrenia. J. Cell. Physiol..

[115] Dube U (2019). An atlas of cortical circular RNA expression in Alzheimer disease brains demonstrates clinical and pathological associations. Nat. Neurosci..

[116] Kong F (2021). RNA-sequencing of peripheral blood circular RNAs in Parkinson disease. Medicine.

[117] Zhong L, Ju K, Chen A, Cao H (2021). Circulating CircRNAs panel acts as a biomarker for the early diagnosis and severity of Parkinson’s disease. Front. Aging Neurosci..

